# First Complete Genome Sequence of a French *Bovine coronavirus* Strain

**DOI:** 10.1128/genomeA.00319-17

**Published:** 2017-05-25

**Authors:** Nathalie Kin, Pauline Guerard, Laure Diancourt, Valérie Caro, Astrid Vabret, Meriadeg Ar Gouilh

**Affiliations:** a Normandie Université, Caen, France; b UNICAEN, UNIROUEN, GRAM, Caen, France; c Department of Virology, University Hospital of Caen, Caen, France; d Institut Pasteur, Environment and Infectious Risks Research and Expertise Unit, Paris, France

## Abstract

We sequenced the first *Bovine coronavirus* (BCoV) complete genome sequence from France. This BCoV was directly sequenced from a fecal sample collected from a calf in Normandy in 2014.

## GENOME ANNOUNCEMENT

*Bovine coronavirus* (BCoV) belongs to the *Nidovirales* order, the *Coronaviridae* family, the *Coronavirinae* subfamily, and the *Betacoronavirus* (https://talk.ictvonline.org/ICTV/proposals/2008.085-122V.v4.Coronaviridae.pdf). Its genome is a single-stranded, linear, and nonsegmented RNA of around 31 kb. BCoV is responsible for respiratory and enteric diseases in cattle, particularly during winter (1, 2). To date, the 19 complete BCoV genome sequences available in GenBank databases (consulted on 17 January 2017) originated from the United States or Asia. Here, we report the first complete genome sequence of a BCoV detected in France.

The BCoV/FRA-EPI/CAEN/2014/13 strain was obtained from a fecal sample collected from a 1-week-old calf in Normandy in 2014. The presence of BCoV in the fecal sample was assessed using an in-house reverse transcription-PCR (RT-PCR) targeting the M gene (3). A cDNA library was synthesized using SuperScript III (Invitrogen, Carlsbad, CA, USA) and hexamers. The complete genome sequencing of overlapping PCR products was carried out in both directions, using original primers and Sanger’s dideoxy sequencing. Sequencing reactions were performed as previously described (3). Sequences were assembled and annotated using the Geneious software (version 5.1.6). We obtained a sequence counting 30,847 nucleotides. The orf1ab, HE, S, ns5, E, M, and N genes of the obtained BCoV were submitted to a Blastn analysis. According to these analyses, the orf1ab (20kb nucleotides, located at the 5′ side of the genome) gene is closely related to the *Dromedary camel coronavirus* (DcCoV) HKU23–23-362F strain from the United Arab Emirates (accession no. KF906251), with a nucleotide identity of 99.19%. Conversely, the NS2, HE, S, ns5, and M genes are closely related to the BCoV Bubalus/Italy/179/07-11 strain (accession no. EU019216), with nucleotide identities of 99.88%, 99.45%, 99.02%, 98.79%, and 99.28%, respectively. The E gene is closely related to the Chinese *Bovine coronavirus* strain BCV-AKS-01 (accession no. KU886219), with a nucleotide identity of 100%. Finally, the highest Blastn score for the N gene was found with the American enteric BCoV-ENT (accession no. AF391541), associated with a nucleotide identity of 100%.

Multiple-sequence alignment, including 20 BCoVs and 10 clade A betacoronaviruses closely related to BCoV from North America, two DcCoVs from the United Arab Emirates, and two *Human coronavirus* OC43 (HCoV-OC43) strains from France, was performed using the Muscle algorithm implemented in MEGA7 (4, 5). The phylogenetic analysis on the orf1ab confirms that BCoV/FRA-EPI/CAEN/2014/13 is closely related to the *Dromedary camel coronavirus* (DcCoV) HKU23–23-362F. The orf1ab gene of these two viruses together clustered separately from that of BCoV and BCoV-like viruses from North America and Asia. This finding also confirms the results from our previous analysis on partial genomes in which nsp12, S, and N genes of American and Asian BCoVs group together in a cluster tentatively named C_1_. The nsp12 and N coding regions of BCoVs from France and DcCoVs from the United Arab Emirates clustered together in C_2_. The DcCoV S gene individualized from both HCoV-OC43 and BCoV S genes. Potential recombination events could be at the origin of DcCoV.

### Accession number(s).

The complete genome sequence sequence of the BCoV/FRA-EPI/CAEN/2014/13 isolate has been deposited in GenBank under the accession number KX982264.
